# Diet and Age Interactions with Regards to Cholesterol Regulation and Brain Pathogenesis

**DOI:** 10.1155/2010/219683

**Published:** 2010-04-11

**Authors:** Romina M. Uranga, Jeffrey N. Keller

**Affiliations:** ^1^Instituto de Investigaciones Bioquímicas de Bahía Blanca, Universidad Nacional del Sur and Consejo Nacional de Investigaciones Científicas y Técnicas, Bahía Blanca, Argentina; ^2^Pennington Biomedical Research Center, Louisiana State University System, 6400 Perkins Road, Baton Rouge, LA 70820, USA

## Abstract

Cholesterol is an essential molecule for brain homeostasis; yet, hypercholesterolemia and its numerous complications are believed to play a role in promoting multiple aspects of brain pathogenesis. An ever increasing number of individuals in modern Western Society are regularly consuming diets high in fat which promote the development of hypercholesterolemia. Additionally, modern societies are becoming increasingly aged, causing a collision between increased hypercholesterolemia and increased aging, which will likely lead to the development of increased pathological conditions due to hypercholesterolemia, thereby promoting deleterious neurochemical and behavioral changes in the brain. Lastly, while beneficial in controlling cholesterol levels, the long-term use of statins itself may potentially promote adverse effects on brain homeostasis, although specifics on this remain largely unknown. This review will focus on linking the current understanding of diet-induced hypercholesterolemia (as well as statin use) to the development of oxidative stress, neurochemical alterations, and cognitive disturbances in the aging brain.

## 1. Overview of Cholesterol

**Figure 1:**
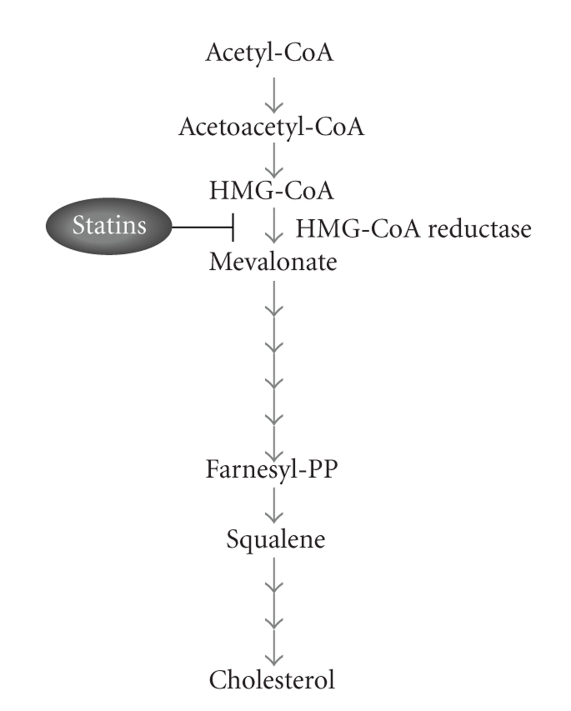
Schematic colesterol biosynthetic pathway. Statins lower cholesterol synthesis by inhibiting 3-hydroxy-3-methylglutaryl coenzyme A (HMG-CoA) reductase.

Cholesterol is an essential component of cell membranes which plays an important role in the maintenance of cellular homeostasis and transmembrane communication within, and between cellular compartments [1]. Cholesterol biosynthesis is a multistep process which takes place mainly in the liver and the intestine, and involves more than twenty enzyme-catalyzed reactions for converting acetate into cholesterol (Figure 1). The rate limiting reaction in cholesterol biosynthesis is catalyzed by 3-hydroxy-3-methylglutaryl-CoA (HMGCoA) reductase, and it is this enzymatic step which has been used as pharmacological target of statin treatments (see below).

Since cholesterol is insoluble in water, it is transported in blood through complex micelle-like amalgamations of various proteins and lipids. These particles, referred to as lipoproteins, are heterogeneous in size, shape, composition, function, and are differentially linked to vascular disease [2]. High-density lipoprotein (HDL) particles, commonly referred to as “good cholesterol”, promote vascular health by taking cholesterol out from tissues and delivering it back to the liver, resulting in the elimination of excess cholesterol from the body. This process is at the center of HDL-mediated prevention of low-density lipoprotein (LDL)-cholesterol build up within arterial walls. Levels of HDL cholesterol below 35 mg/dL represent a substantially higher risk for coronary heart disease [3, 4], and therefore maintaining HDL cholesterol levels about 35 mg/dL is necessary to maintain overall health. LDL particles, also referred to as “bad cholesterol”, carry most of the cholesterol in the circulation and tend to be readily oxidized and bind to connective tissue in the intimal sublayer of arteries [5, 6]. Thus, the higher LDL cholesterol in blood, the greater the risk of subsequent cardiovascular disease. Before reaching a particle of the HDL or LDL subfraction, cholesterol molecules will usually undergo a maturation process beginning with the hepatic or intestinal synthesis of very low density lipoprotein (VLDL) particles. These lipoproteins not only contain cholesterol but also contain fatty acids (esterified to triglycerides), phospholipids, and apolipoproteins [2].

## 2. High Fat Diets and Age-Related Changes in Cholesterol Metabolism

The effects of high fat diets (HFDs) on cholesterol metabolism have been studied in both humans and animals. HFD consumption has been associated with hyperlipidemia. Studies performed in humans have shown that HFD consumption causes an increase in both total and LDL cholesterol levels in plasma, a significant reduction in HDL cholesterol, and an increased total cholesterol = HDL cholesterol ratio [7]. Erythrocyte and leukocyte membranes were observed to have increased cholesterol and phospholipid content following HFD consumption, and demonstrated to have an elevated cholesterol = phospholipid molar ratio [7]. Interestingly, these metabolic alterations are reversed slowly after returning to a normal (low-fat) diet.

A clear increase in plasma cholesterol levels has been found in all mammals, including humans, during the suckling period [8]. An increase, as well as a decrease, in the levels of blood cholesterol has also been observed following weaning depending on the diets consumed postweaning [9]. Taken together with many studies carried out in different populations, it is clear that plasma lipid levels in adults are attributable to the quality and quantity of dietary lipid intake. People consuming a diet that is balanced and low in fat exhibit low total cholesterol and low LDL cholesterol levels [10–12]. However, when these people are placed on more typical Western diets (high in fat), their total and LDL cholesterol levels increase [10, 13].

It is believed that cholesterol turnover is strongly correlated with body fat. For example, it has been shown that cholesterol turnover is higher in obese individuals as compared with nonobese subjects, and that the excess of body fat correlates significantly with the daily production of cholesterol [14]. It is thought that different levels of cholesterol and adiposity following HFD consumption could be due to adipocyte number, cell membrane phospholipids and glycoproteins, and cholesterol-esterifying activity in cells, all of which are capable of modulating adiposity and cholesterol levels following HFD consumption [7]. Long-term HFD consumption leads to a significant increase not only in body weight but also an increase in serum concentrations of triglycerides and cholesterol [15]. Interestingly, studies carried out in hypercholesterolemic rabbit model of atherosclerosis and Alzheimer's disease (AD) have demonstrated that high-cholesterol diet is able to induce significant increases in plasma and liver cholesterol, but did not significantly alter brain cholesterol levels [16]. It is not yet fully known how cholesterol may modulate the susceptibility to neurological disorders. It is well known that high cholesterol levels in plasma are sufficient to promote atherosclerosis in large arteries [17] and there is also evidence showing that high cholesterol adversely affects the function of the microvasculature, including microvessels in the brain [18]. In this regards, Franciosi and coworkers have reported that mice fed high-cholesterol diet develop a vascular pathology that affects the central nervous system (CNS) microvasculature [19]. This vascular pathology seems to be different from cholesterol-induced atherosclerosis and shares some features with the microvascular pathology commonly associated with AD [20]. Further studies on this issue are important for clarifying the role of cholesterol in mediating the vascular pathology observed in neurodegenerative diseases such as AD.

When discussing cholesterol disturbances in relation to age-related diseases of the nervous system, it is important to consider aging-induced changes in cholesterol metabolism. A number of metabolic changes have been reported to occur during normal aging in both animals and humans [21]. Reduced physical activity, redistribution of body tissues with a relative increase in adipose over muscle mass [22], increased insulin resistance [23, 24], and increased blood pressure [25] are all factors that could contribute to the acceleration of age-related atherosclerosis. Plasma levels of total and LDL-cholesterol are well known to increase with normal aging both in humans and rodents [26–29], and the plasma clearance of LDL has been shown to decrease with age in both humans and rodents [26, 28, 30], with high plasma LDL-cholesterol levels being key causal factors for the onset of atherosclerosis and coronary heart disease. This observation may be related to the decreased breakdown of cholesterol to bile acids reported to occur in aging rodents [29, 31], as well as to the increased intestinal cholesterol absorption in aging mice and rats [31, 32]. Additionally, it has been reported that cholesterol metabolism is markedly modified during normal aging [33], and in humans plasma LDL-cholesterol increases by about 40% from 20 to 60 years of age [34]. Important to point out is the role of growth hormone in cholesterol metabolism. It has been shown that growth hormone has key roles in lipid metabolism in adult humans and animals [35–37], particularly in some steps of cholesterol metabolism. In this regards, it has been reported that the secretion of growth hormone is reduced with aging [21, 38], and experiments performed in rodents have demonstrated that the administration of growth hormone is able to completely reverse the age-dependent increase in plasma cholesterol, as well as the reduced level of bile acid synthesis to the same levels as seen in young animals [29]. Taken together, these data clearly suggest that the decreased secretion of growth hormone during aging may be a causal factor contributing to the age-dependent rise in serum cholesterol.

## 3. Cholesterol, Cholesterolemia, and the Brain

Cholesterol is a well-known key regulator of membrane lipid organization and fluidity, and thus homeostatic mechanisms to maintain cellular cholesterol levels in membranes are essential for life. Even though the CNS represents only 2.2% of body weight, it contains as much as 23% of total body cholesterol [8]. Interestingly, the half-life of brain cholesterol is 6 months–5 years [39–41], in contrast to its half-life in plasma which is measured in hours [1, 8, 42–46].

The synthesis of cholesterol in the brain occurs mainly in olygodendrocytes and astrocytes [42]. While it is well known that oligodendrocytes generate cholesterol for the myelination process, it has been shown that astrocytes produce cholesterol for neuronal cells, using a transport system that involves ATP-binding cassette A1 (ABCA1) and ATBCG1 transporters [47–49]. These transporters are expressed in neurons, astrocytes and microglia as well, and are necessary for maintaining the physiological levels and production of lipoproteins secreted by astrocytes and microglia [49, 50]. Active axonal growth, as well as synapse formation and remodeling, requires cholesterol that cannot be provided by the distant cell body of the neuron. Cholesterol for these processes may be derived primarily from astrocytes, via the synthesis and secretion of ApoE-associated cholesterol at sites near the axonal growth and synapse formation [51].

It has been suggested that neurons, during development, are able to synthesize most of the cholesterol they need for growth and synaptogenesis [46]. However, as soon as they mature, they have an impairment in this process and grow to depend on cholesterol provided by astrocytes [1, 52]. This reduction in endogenous neuronal cholesterol biosynthesis in adult brains likely results from the large metabolic requirement for the biosynthesis of cholesterol, and the need for optimal energy efficiency within the CNS. Interestingly, it has been shown that brain-derived neurotrophic factor (BDNF) is an important stimulus for neuronal *de novo* synthesis of cholesterol [53]. Since cholesterol has structural features that make membranes more rigid, maintaining the proper level of cholesterol is essential for neuronal membrane function, especially at the synaptic endings, where membrane fluidity influences the process of neurotransmission [54].

The cholesterol resident within the brain exists in two pools. The major pool accounts for approximately 70% of the total brain cholesterol, is metabolically stable, and is found in the myelin membranes of white matter [55, 56]. The concentration of cholesterol in this pool is about 40 mg/g tissue and reflects the dense packing of multiple opposed lipid bilayers in the myelin sheath [57]. The second pool of cholesterol is less abundant, about 30% of total cholesterol pool, and is found in the plasma and subcellular membranes of neurons and glial cells within the grey matter [57]. The concentration of cholesterol in this pool is lower (8 mg/g), and it is metabolically active and in an unesterified form.

Many of the same proteins involved in the movement of cholesterol throughout the body are also expressed in the CNS including many members of the LDL receptor family as well as scavenger receptor class B type I (SR-BI) [58, 59]. The apolipoproteins ApoE, ApoA-I, ApoA-IV, ApoD, and ApoJ have also been reported in cerebrospinal fluid (CSF), and various members of the ABC family of transporters are expressed in specific cells of the CNS [60, 61]. Interestingly, any lipoprotein-carried cholesterol that enters or leaves the CNS must cross the blood brain barrier (BBB), which anatomically is made up of unique endothelial cells whose basal membranes are also intimately associated with the foot processes of adjacent astrocytes [62]. Capillaries of the brain appear to have no fenestrae and show very little capability for bulk phase vesicular transport [8]. Moreover, adjacent endothelial cells are tightly adherent, so it is very unlikely that cholesterol carried in lipoproteins can reach the CNS either through fenestrations in the capillary membranes or through paracellular diffusion [63, 64]. From this point of view, it would appear that circulating cholesterol has a minimal contribution to levels of cholesterol in the adult brain. However, several studies have determined that circulating levels of cholesterol do have direct consequences on the brain. For example, a physiological hypercholesterolemia is observed during suckling [8], and this is at the time of major growth and myelination in brain, so restrictions in diet cholesterol are thought to be deleterious for later brain development and performance [65, 66]. Moreover, many investigations have reported that adults with low-blood cholesterol exhibit aggressive, suicidal, or criminal behavior [67–70], and the possible cause could be a reduction in the number of serotonin receptors in brain as a consequence of a loss of cholesterol in membranes [71]. In addition, it has been found that there is a positive correlation between plasma cholesterol levels and the incidence of dementias and AD [72–74]. Taking into account that plasma lipoprotein-carried cholesterol pool does not cross the BBB, the question is how would cholesterolemia have consequences in brain performance? Firstly, it is possible that plasma membrane of endothelial cells contain functional lipoprotein transporters or transporters for unesterified cholesterol like ABCA1. Secondly, it is possible that a small amount of bulk-phase endocytic transcellular movement could take place in endothelial cells. Thirdly, it has been demonstrated that the BBB is permeable to some hydrophobic molecules such as sterols and that hydroxylation of sterols greatly increases their rate of passive diffusion across the BBB [8]. In fact, brain 27-OH cholesterol has been found to correlate with plasma cholesterol levels, so it is believed to maintain cholesterol homeostasis within the brain [40, 41, 75]. In this regard, it is possible that unesterified or hydroxylated cholesterol could diffuse passively across the BBB through existing gradients [8, 76]. Further research is necessary to address this issue and to clarify the mechanisms by which levels of circulating cholesterol “communicate”/relate with those present in brain.

## 4. ApoE and Brain

ApoE is one of the major apolipoproteins in plasma and the principal cholesterol carrier protein in the brain. In recent years, brain cholesterol and ApoE have been intensively explored in relation to brain aging and age-related diseases of the brain. Studies with transgenic mice lacking ApoE (ApoE^−/−^) have demonstrated that the loss of ApoE increases behavioral deficiencies, oxidative stress, synaptic dysfunction, and enhanced pathogenesis following brain injury [77–80]. Taken together, these data clearly implicate ApoE in the regulation of brain pathogenesis.

While plasma ApoE originates predominantly from the liver and macrophages, the brain is able to locally synthesize it, and plasma pool of ApoE does not appear to readily exchange with the brain pool owing to the presence of the BBB [8, 81, 82]. Astrocytes are the main ApoE source in brain, although neurons and microglia are also able to synthesize it in some pathological conditions [44, 83–85]. Astrocytes secrete ApoE in discoidal HDL particles composed of phospholipids and unesterified cholesterol. It appears that before reaching the CSF some of these particles accumulate cholesterol and form spherical lipoproteins upon esterification of free cholesterol [86].

ApoE in the brain is associated to the only lipoproteins found in the CNS, the HDL-like lipoproteins [60, 87–89]. Other apolipoproteins are also found in the brain: ApoA-I, ApoA-II, ApoA-IV, ApoD, ApoE, ApoH, and ApoJ, being ApoE the predominant [90]. ApoE is expressed in several organs, with the highest expression in the liver, followed by the brain. Interestingly, ApoE-containing lipoproteins in the CSF are produced predominantly, if not exclusively, by cells within the CNS [82]. Although neurons are able to produce ApoE in some situations, it is mainly expressed by astrocytes and to some extent microglia [84, 87, 90–95].

In humans, ApoE gene contains several single-nucleotide polymorphisms distributed across the gene [96]. The most common three polymorphisms result in the three common isoforms ApoE2, ApoE3, and ApoE4, and although they differ only in one or two aminoacids, they deeply differ in structure and function [97]. Several studies have shown that *ε*4 allele is a strong risk factor for both AD and cerebral amyloid angiopathy (CAA), whereas the *ε*2 allele is associated with decreased AD risk [98–102]. It has been suggested that the effect of ApoE isoforms on AD and CAA would be mediated by interactions between ApoE and the amyloid-*β* (A*β*) peptide [103], altering the peptide's clearance and fibrillogenesis [104–107]. Based on the strong association between ApoE and A*β* in the brain [108], it was hypothesized that ApoE may function as an A*β*-binding protein that induces a pathological *β* sheet conformational change in A*β* [102]. It is not known yet if the ApoE2 protective effect is due to modulation of A*β* aggregation or to an alternative mechanism independent of amyloid plaque formation [109]. Most studies have demonstrated that the efficiency of complex formation between ApoE and A*β* is ApoE2 > ApoE3 ≫ ApoE4 [110]. As these data represent an inverse correlation with the risk of developing AD, it has been suggested that ApoE2 and ApoE3 may enhance the clearance of A*β*, compared to ApoE4. It has also been shown that the three isoforms of ApoE promote A*β*42 fibrillization, with the maximal fibrillization occurring with the ApoE4 isoform, and minimal with ApoE2 [111]. Other studies using transgenic mice that possess different human ApoE transgenes have also demonstrated that A*β* deposition occurs in an isoform-dependent manner (E4 > E3) [112, 113]. Interestingly, experiments performed in Tg2576 mice, which have human ApoE3 and ApoE4 as knock-in genes, have shown that both human ApoE isoforms delayed the onset of A*β* deposition relative to control mice not having the human genes, with human ApoE4 promoting more amyloid deposition and CAA than human ApoE3 [114]. Important to note, complexes of A*β* and ApoE2 or ApoE3 are cleared out of the brain significantly faster than complexes of A*β* and ApoE4 [115]. All these data clearly support the idea that ApoE promotes A*β* deposition, with ApoE4 promoting the most severe effect. However, these studies do not exclude the possibility that all three ApoE isoforms are inhibitors of A*β* aggregation, with ApoE4 being the least effective [100].

## 5. Cholesterol and Synaptic Function

Aging is the major risk factor not only for AD but for other neurodegenerative disorders as well. Therefore, before moving on to discussing the roles of cholesterol and ApoE in the pathophysiology of neurodegeneration, it is worth spending a moment to understand the changes in cholesterol and ApoE during normal aging. Many studies have reported the decrease in weight and size of human brain during aging [116], and it is suspected that these changes are due to reduction in neuronal size and in synapse number, both parameters associated with cognitive impairment [117]. Brain cholesterol levels do not change in a uniform manner in the brain, instead, the changes vary according to the region studied. Some regions do not show any change while others show a decrease of 40% [118]. It has been also reported that there is a decrease in the content of cerebral cortex cholesterol throughout life, and this decrease would represent the loss of axons and processes in the cerebral cortex [119].

As stated above, cholesterol is crucial for synapse generation, since it increases the number of synaptic vesicles, which contain high levels of cholesterol [120–122]. Additionally, cholesterol is considered to be essential for remodeling neuronal membranes and growing new terminals, either during synaptic plasticity or in response to a neurodegenerative insult. Moreover, if cholesterol is not supplied by astrocytes, neurotransmission is deleteriously affected. It has been suggested that the molecular basis of this phenomenon is related to the failure in the formation of lipid rafts in the absence of cholesterol [51]. Lipid rafts are believed to be essential for intracellular signaling, as they concentrate the signaling pathways at membranes, and they seem to be particularly important at the synapse level. In this regard, it has been reported that the absence of cholesterol causes loss in synaptic endings and dendritic spines [51]. So it could be speculated that the cognitive decline observed during aging could be related to the decrease in synaptic transmission, and this could be associated, at least in part, with the age-related decrease of brain cholesterol. On the other hand, cholesterol has been shown to enhance the efficacy of presynaptic transmitter release, enable dendrite differentiation, and promote the redistribution of glutamate receptors [123]. Additionally, as neurons are not able to produce sufficient amounts of cholesterol on their own, they may need cholesterol provided from astrocytes as building material for dendrites and synapses [124].

In addition, it has been reported that ApoE levels might be important for brain homeostasis during aging. Although it is not clear whether ApoE expression in the brain changes with aging, some studies in rodents have shown that the expression of this apolipoprotein is decreased in hypothalamus and cortex [125], however, it is increased in the hippocampus [126]. It is suspected that ApoE plays a key role in spatial learning and memory process, since studies carried out in ApoE-null mice have shown that infusion of ApoE via the intracerebroventricles is able to revert the memory and spatial learning impairment caused by the lack of ApoE [127].

In regards to synaptic plasticity, many studies have been carried out in ApoE knockout (KO) mice. In vivo neurophysiological studies in the hippocampus of aged ApoE KO mice have shown that the absence of ApoE generates a decrease in long-term potentiation [128]. Moreover, Krugers and coworkers have also reported altered synaptic plasticity in ApoE KO mice, with deficits in long-term potentiation [129]. All these evidence clearly suggest that behavioral and neuropathological alterations observed in ApoE KO mice could be related to alterations in synaptic plasticity. The mechanism by which ApoE would alter synaptic plasticity remains unclear, although some recent evidence suggests that it would be related to calcium regulation [130]. In addition, ApoE2 overexpression has been shown to protect against dendritic spine loss reported to occur in the hippocampus of young amyloid precursor protein (APP)-transgenic mice. Many in vivo studies have shown that ApoE3 is able to increase synaptic plasticity and exert neuroprotective effects, whereas results about ApoE4 effects on synaptic plasticity are inconsistent. Some studies say that ApoE4 has a negative effect on neurites and synaptic functions [131] and others propose that it may have beneficial effects [127, 132]. Further studies would be needed to clarify this issue.

## 6. Cholesterol and Cerebral Blood Vessels

It has been shown that hypercholesterolemia can result in the damage to endothelial cells of arteries and capillary vessels, decrease in blood flow, impairment of metabolism, and the decrease in nutritive and oxygen levels in the brain [133], thus increasing the possibility of cognitive impairment. It is well known that hypercholesterolemia and hypertension are two of the most common age-related metabolic disturbances. It has been reported that hypercholesterolemia and/or hypertension impair endothelial function in peripheral vasculature. However, their impact on endothelial cells of brain microvessels is unclear. Some studies have shown that hypertension would increase BBB permeability, and the extent of that increase has been shown to be smaller in hypercholesterolemic animals with acute induction of hypertension than that observed in chronically hypertensive animals [134]. In addition, it has been reported that subjects with familial hypercholesterolemia have at least 20 times higher risk of brain infarction than in the general population, so subjects with familial hypercholesterolemia have not only a high risk of coronary heart disease but also a high risk of cerebrovascular disorders [135]. On the other hand, alteration of cerebral blood flow and hypoperfusion of brain regions due to arteriosclerosis of the cerebral vasculature might precede frank dementia by many years [136]. In this regard, it has been reported that even small asymptomatic cerebral infarcts can increase the probability of expressing clinical symptoms of dementia by 20 times in individuals with existing senile plaques and neurofibrillary-tangles [137].

**Figure 2:**
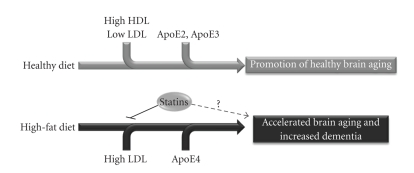
Factors that influence in the progression of normal and pathological brain aging. The scheme shows how the interaction between different dietary and genetic factors (ApoE alleles) leads to different final outcomes in regards to brain aging.

An additional link between cholesterol and AD is that cholesterol has been also associated with CAA, a highly prevalent disorder in AD where A*β* deposits in cerebral blood vessel walls. It has been suggested that ApoE-cholesterol-lipoprotein complex would be involved in the process of A*β* deposition. ApoE *ε*4 allele has been strongly associated with the increase in vascular deposition of A*β*, the formation of neuritic plaques, and the development of the CAA pathology in mouse models of AD [138–140] (Figure 2). All these data suggest that cholesterol is implicated in the development of AD and vascular dementia, though the mechanisms involved remain elusive.

## 7. Cholesterol and Neuropathology

Several different studies have provided converging evidence for a link between vascular injury and AD in the brain. AD and vascular dementia have been demonstrated to be coexisting processes that contribute to the expression of dementia [141]. Hypertension, diabetes mellitus, and hypercholesterolemia are generally associated with a high risk of developing AD [141–144]. Additionally, a growing body of evidence suggests a connection between cholesterol metabolism and susceptibility to AD, as well as increased cholesterol release through synaptic degeneration [145–148]. In rat hippocampal neuron cultures, reduction of intracellular cholesterol levels has been shown to inhibit the production of A*β* [149]. Studies with double transgenic (APP-presenilin) mice have shown that high dietary cholesterol increases A*β* accumulation [150], and cholesterol lowering agents increase processing of APP through the nonamyloidogenic *α*-secretase pathway in different cell lines via increased membrane fluidity [151]. Moreover, animals fed high-cholesterol diets have shown to have increased brain A*β* levels which are reduced when animals return to normal chow diets [74]. It has been hypothesized that cholesterol may influence the A*β* metabolism via indirect effects on vasculature, since high plasma cholesterol may contribute to the development of AD via hypoperfusion of the brain promoting the elevated production of A*β* [152–154], followed by cerebrovascular degeneration and CAA. Furthermore, high plasma concentrations of cholesterol in midlife, as opposed to later life, may determine the risk of developing late onset AD. Mediterranean diet (i.e., intake of fish omega-3 polyunsaturated fatty acids, olive oil and unrefined foods, moderate intake of red wine which contains polyphenols and other antioxidants, and low intake of hydrogenated fats) has been associated with reducing the risk of developing AD and other types of dementia, but it has not been defined yet whether this effect is by the diet-induced decrease of plasma LDL or through additional properties of these kind of lipids, such as anti-inflammatory properties [155]. It has been shown that docosahexanoic acid may have a protective effect by maintaining vascular health [156–158]. Other studies have shown that a high-cholesterol environment promotes a reduced production of soluble APP [151, 159]. The molecular basis by which cholesterol affects A*β* production is not clear yet. It has been suggested that cholesterol might cause changes in membrane properties which could affect membrane-bound enzymes such as those that produce the A*β* [160, 161].

The etiology of Parkinson's disease (PD) and related diseases such as dementia with Lewy bodies (DLB) is poorly understood, although epidemiologic evidence suggests that dietary factors may contribute to the development of PD [162]. Dietary fats, including cholesterol, have been associated with PD in some studies and are believed to contribute to oxidative stress [163]. However, other studies have also shown either no association [164–166] or an increased risk for PD based on dietary fat [167]. Surprisingly, high-plasma cholesterol levels have recently been related to a decreased PD risk [168, 169]. As discussed previously in this review, ApoE *ε*4 allele represents a high-risk factor for AD. However, a high prevalence of the ApoE *ε*4 allele, with its cholesterol disturbances associated, has also been found in vascular dementia and DLB [170–172]. The possession of this allele is associated with high plasma levels of total cholesterol and LDL-cholesterol, both known to be risk factors for developing atherosclerosis, and it is thought that atherogenesis together with neurodegenerative mechanisms may influence the development of dementia [173] (Figure 2).

## 8. Cholesterol and Neuroinflammation

It is now known that cholesterol-related disorders such as atherosclerosis as well as AD and many other neurodegenerative diseases all involve inflammatory processes. Atherosclerosis-related inflammation involves changes in the endothelium provoked by oxidized lipids and lipoproteins, monocyte and macrophage accumulation, and finally sclerotic plaque formation [174]. Inflammatory processes have also been linked to AD since cytokines and other inflammatory mediators have been found in vulnerable regions of AD brains [175–177]. The influence of high cholesterol levels in plasma on cognitive performance has been studied in mice, particularly from the point of view of neuroinflammation and APP processing. It has been found that high-fat/high cholesterol diet impairs working memory in normal mice and induces neuroinflammation with glial activation and increased expression of cytokines and oxidative stress-related enzymes (TNF*α*, IL-1*β*, IL-6, iNOS and COX2) [178]. Importantly, neuroinflammatory changes in the hypercholesterolemic mice are closely correlated with behavioral changes so it has been suggested that cholesterol-induced neuroinflammation may play a causal role in memory dysfunction [178]. It has been shown that cholesterol is able to induce A*β* production; however, it is thought that cholesterol-induced neuroinflammation would be more related to vascular alterations than to the production of A*β* itself. It has been hypothesized that high cholesterol levels in plasma may cause cerebrovasculature dysfunction thus triggering the activation of perivascular microglia with scavenger functions in the brain [179]. It is thought that the recruitment of immune cells to the inflamed brain arterioles may hence initiate a series of events leading to an altered amyloid processing, neurodegeneration, and synaptic and cognitive dysfunction. In addition, it is believed that high cholesterol levels in plasma may induce vascular changes as those observed in early inflammatory lesions of atherosclerosis as well as an increase in BBB permeability. Studies in rabbits have shown that cholesterol is able to induce A*β* and iron deposition in cerebral cortex together with BBB disruption [180]. It has been also suggested that inflammatory stimuli are able not only to provoke neurotoxic and synaptotoxic effect but also to induce amyloid generation, since up-regulating BACE1 [181, 182]. On the other hand, APP metabolism itself seems to regulate cholesterol homeostasis through the regulation of the expression of LDL receptor-related protein-1 [183]. Interestingly, it appears that AD brains do not contain increased levels of cholesterol [184].

## 9. Cholesterol and Cerebral Oxidative Stress

Several studies have shown that high fat/high caloric diets which lead to hyperlipidemic states increase the free radical generation [185] and protein oxidation in the brain of rodents [186]. Additionally, they clearly suggest a relationship between AD and hypercholesterolemia, in addition to coronary artery disease and hypertension [187]. It has been reported that hypercholesterolemia increases the levels of reactive oxygen species so it is possible that hypercholesterolemia facilitates the disease development of the neurodegenerative disease through increased oxidant production [188].

Reactive oxygen species are normal byproducts in cells during aerobic metabolism [189], whose generation is tightly controlled, but when overproduced then a phenomenon of oxidative stress is observed [190, 191]. Reactive oxygen species are known to interact with proteins, lipids, and nucleic acids, modifying not only their structure but their functions as well [192]. A great body of evidence has demonstrated that high-fat diet-induced hyperlipidemia has tight relations with vascular damage and oxidative stress [15]. Hyperlipidemia has been shown to incite endothelial cell activation, lipid deposit, and oxidative stress, throughout the microvasculature [193, 194]. Both experimental and clinical studies have suggested that oxidative stress is involved in the pathogenesis of a number of diseases [194–197] and that high-fat diets promote liver injury and insulin resistance through oxidative stress [198–200], with increased production of reactive oxygen species causing lipid peroxidation followed by inflammatory response [201, 202]. Studies performed in rabbits fed high-cholesterol diets have shown an increase in several markers of oxidative stress, especially in hippocampus, suggesting that hypercholesterolemic diet is able to induce neuropathological-oxidative changes in the brain [188].

## 10. Statins

The role of hypercholesterolemia in the pathogenesis of coronary heart disease has been clearly established, and there is a great body of clinical and epidemiological evidence suggesting that lowering cholesterol with HMG-CoA reductase inhibitors (“statins”) reduces coronary events. As previously mentioned, statins act mainly by inhibiting HMG-CoA reductase, the rate-limiting enzyme in cholesterol synthesis. This inhibition leads to an up-regulation of hepatic LDL receptor and concomitant removal of total and LDL cholesterol from plasma [203]. Lowering circulating cholesterol creates a cholesterol gradient across the BBB so an efflux of cholesterol in the form of 24S-hydroxycholesterol occurs from the brain.

However, statins also seem to have several different mechanisms of action that may contribute to their clinical benefit. Statins have been shown to inhibit accumulation of cholesterol in macrophages in vitro [204], reduce macrophage foam cell formation [205], and inhibit cholesterol accumulation in vascular smooth muscle cells [206]. Additionally, statins have been shown to modify platelet activity in hypercholesterolemic patients [207, 208], an important feature since patients with high LDL cholesterol levels show increased platelet aggregability. Many studies have also suggested that statins, in addition to their role in prevention of vascular events as mentioned previously, have further mechanisms of action that may modify brain injury before, during, and after cerebral ischemia. One of the mechanisms would be the suppression of nitric oxide production, a well-known component of the inflammatory response [209]. Many cytokines are produced by neurons, glia, and endothelium as mediators of inflammatory response in the brain, and statins have been shown to reduce the elaboration of these potentially detrimental cytokines from macrophages [210]. Indeed, statins have been shown to inhibit neutrophil adhesion to coronary endothelium [211] and reduce leukocyte-endothelium interactions in hypercholesterolemic animals [212]. A number of studies have suggested that statins reduce lipoprotein oxidation and ameliorate free radical injury, both of which have been found to be implicated in brain injury, particularly during reperfusion after an ischemic event.

As cholesterol levels have been related to AD development, many studies have identified statin treatment as a potential AD therapy. Some studies have shown statins to be associated with increased activity of alpha secretase and decreased concentrations of extracellular A*β* [213]. Another interesting point is that statins may also modify the synaptic plasma membrane distribution of cholesterol with reduction in the cholesterol content of the exofacial leaflet [214]. By stabilizing the cholesterol distribution in the membrane leaflet, statins may prevent cleavage of APP into pathological A*β* isoforms [214]. Two epidemiological studies investigating the prevalence of dementia among statins users have shown that statins are associated with a lower risk of dementia, with one study reporting a relative risk reduction [215, 216]. However, although statin treatment seems to be beneficial for lowering cholesterol levels, potential adverse effects have been attributed such as increased risk of intracerebral hemorrhage and cognitive impairment [217]. On the other hand, several animal studies have also reported potential neurotoxicity with statin use [218, 219].

## 11. Conclusions

There are clear direct and indirect roles for cholesterol in regulating the development of age-related dementia. Treatment with statins is not a clear solution to preventing the deleterious effects of high cholesterol on the brain, as chronic statin use may adversely affect the aging brain. Understanding the mechanisms by which cholesterol and statins mediate their effects on brain is therefore a growing and important area of future research.
